# Mandibular extracellular vesicles mediate morphogenesis and mineralization of tooth germs in miniature swine through the miR-206/HDAC4 signaling axis

**DOI:** 10.3389/fcell.2025.1707072

**Published:** 2026-01-05

**Authors:** Xiaoyu Cao, Duanlin Ma, Yujiao Song, Yiping Gao, Wen Liu, Xiaohong Du, Xiaojun Sun

**Affiliations:** 1 Department of Oral Medicine, First Hospital of Shanxi Medical University, Taiyuan, Shanxi, China; 2 Shanxi Medical University and Hospital of Stomatology, Taiyuan, Shanxi, China

**Keywords:** tooth development, mandible, miR-206, HDAC4, epigenetic regulation

## Abstract

**Background:**

Reciprocal communication between odontogenic tissues underpins the complexity of tooth morphogenesis. Despite mandible serving as the developmental niche and functional platform for tooth germs, their reciprocal signaling mechanisms remain underexplored. Histone acetylation plays a pivotal role in maintaining long-term regulatory equilibrium and physiological homeostasis by establishing stable gene expression patterns. However, whether stable histone acetylation signatures exist during tooth germ morphogenesis and how they might ensure developmental fidelity remain unreported.

**Methods:**

Extracellular vesicles were isolated from E40 miniature pig mandibles, with bioinformatic analysis identifying miR-206 as a key miRNA targeting the epigenetic regulator HDAC4. Mechanistic validation utilized dual-luciferase reporter assays, qRT-PCR, and Western blotting to confirm target interactions *in vitro*. *In vivo* assessment, tooth germs were co-cultured with mandibular EVs or lentivirally transduced for miR-206/HDAC4 overexpression/knockdown, then subcutaneously transplanted into nude mice. Harvested tooth germs underwent stereomicroscopic morphological analysis, micro-CT-based 3D reconstruction with mineralization quantification, and H&E histogenesis evaluation to validate the miR-206/HDAC4 regulatory axis.

**Results:**

*In vivo* and *in vitro* findings collectively validated miR-206 as the critical regulator within mandibular-derived extracellular vesicles. Exosomal miR-206 suppressed HDAC4 expression in tooth germs, epigenetically mediating morphogenesis and mineralization during early stage of tooth development.

**Conclusions:**

Our identification of the exosomal miR-206/HDAC4 signal axis redefines the mandible as an active epigenetic modulator of odontogenesis. This vesicle-mediated regulation enables long-range delivery of epigenetic effectors—revealing a paradigm shift in tooth development and a druggable target for tooth regeneration.

## 1 Introduction

Tooth loss negatively impacts oral function, aesthetics, and overall quality of life. It can lead to systemic issues, such as nutritional deficiencies, and is associated with mental health problems (Xu et al., 2023; Blazer, 2022). Despite restoring part of masticatory function, current prosthetic solutions for tooth loss fail to provide vascularization and innervation, which are essential for full physiological function (Zhang and Yelick, 2021). Compared with these, tooth regeneration presents a superior alternative strategy, positioning it as a central pursuit in dental and regenerative medicine (Duailibi et al., 2006). Despite various strategies for whole-tooth regeneration, none fully replicate the structural hierarchy and functionality of natural teeth, underscoring the necessity of developing functional regenerative strategies (Morsczeck, 2023; Sui et al., 2025).

To effectively regenerate tooth with full functionality and morphology resembling natural tooth, it is crucial to delineate the intricate spatiotemporal regulatory mechanisms that orchestrate odontogenesis. Reciprocal communication between odontogenic tissues underpins the complexity of tooth morphogenesis (Novacescu et al., 2025). Traditionally, previous studies on tissue crosstalk in odontogenesis have been largely confined to interactions within the odontogenic epithelium and mesenchyme (Sui et al., 2023; Zhang et al., 2025; Slavkin et al., 1984). Despite sharing a common embryonic origin in the first pharyngeal arch—with the jawbone providing the developmental niche and functional platform for the tooth germ—their reciprocal signaling mechanisms remain underexplored (Plavcan and Daegling, 2006). Accumulating evidence (Denaxa et al., 2009; Peters et al., 1998) reveals that genetic perturbations during early mandibular ontogeny disrupt reciprocal signaling essential for tooth germ patterning. Nevertheless, there is a persistent critical knowledge gap regarding how the jawbone, as a dynamic niche, orchestrates signaling to direct tooth germ patterning.

To bridge this gap, we employed the miniature pig model—a strategic choice that offers superior clinical relevance over rodent models for studying tooth development. The miniature pig’s diphyodont dentition, complex crown morphology, and patterns of tooth eruption and replacement closely mirror human odontogenesis (Wu et al., 2020; Wang et al., 2007). Beyond these morphological advantages, the miniature pig model provides superior predictive value for therapeutic development. Its comparable jaw size and biomechanical environment (Mehl et al., 2025) to humans offer critical insights for future regenerative strategies, particularly for scaling treatment approaches such as EV-based therapies.

Epigenetic regulation plays a pivotal role in maintaining long-term regulatory equilibrium and physiological homeostasis by establishing stable gene expression patterns (Shen et al., 2015). Among these, histone acetylation—a well-characterized and relatively stable epigenetic modification—plays critical roles in tooth development (Du et al., 2025). It serves not only as a key regulator of mineralization processes in bone (Jiang, 2024), periodontal ligament and dental pulp (Yamauchi et al., 2024), but also emerges as a viable candidate for therapeutic intervention for dentofacial pathologies (Martins et al., 2016; Wang X. et al., 2025). For instance, the odontogenic capacity of human dental pulp cells (hDPCs) is mediated by KAT3B, which epigenetically activates the expression of DSPP and OCN through specific promoter binding and consequent H3K9 hyperacetylation (Krishnan et al., 2022). KAT2A governs murine craniofacial cartilage development, and its downregulation specifically activates the Wnt pathway, thereby modulating osteogenic potential in periodontal ligament stem cells (PDLSCs) (Li et al., 2024). Additionally, histone deacetylases (HDACs) exhibit spatiotemporally restricted expression profiles across mineralized tissues (bone, dentin, cementum), suggesting finely tuned regulatory functions during hard tissue formation (Yamauchi et al., 2020). Furthermore, miRNAs orchestrate developmental (Duckett et al., 2025), immune (Vijaykrishnaraj et al., 2023), and metabolic (Hase and Banerjee, 2025) processes by suppressing epigenetic enzymes (e.g., HDACs, HATs), thereby modulating downstream gene transcription. For instance, Yu et al. (2024) have noted that miR-30b-5p carried by extracellular vesicles from Schwann cells exerts its pro-regenerative effects by directly modulating the Sin3a/HDAC4 complex. This inhibition of HDAC4 relieves its repressive function, thus triggering the phosphorylation of downstream effectors ERK, STAT3, and CREB, which ultimately drives nerve regeneration. However, whether stable histone acetylation signatures exist during tooth germ morphogenesis and how they might ensure developmental fidelity remain unreported.

In this study, we uncovered a novel mechanism of crosstalk between the tooth germ and jawbone to decode dental development. We identified that the mandible secretes extracellular vesicles (EVs) carrying key regulators, which orchestrate the tooth germ patterning through spatiotemporal epigenetic reprogramming. Our findings established the mandible as an active regulator of odontogenesis, providing insights for preventing dental developmental anomalies, and proposed EVs-based innovative strategies for functional tooth regeneration.

## 2 Materials and methods

### 2.1 Harvesting of tooth germs and mandibles

The first deciduous molar (DM1) and mandibles were harvested from E40 miniature pig embryos. This stage was selected based on preliminary co-culture experiments which demonstrated that E40, the early bud stages of DM1 (Stembírek et al., 2010), constitutes a critical window during which the mandible is essential for normal tooth germ morphogenesis (Supplementary Figures S1A–C). E40 pregnant miniature pigs were anesthetized after weight assessment. Using a layered incision technique, abdominal tissues were sequentially dissected. Fetuses were aseptically extracted, rinsed with saline to remove blood residues, and decapitated. Heads were placed in sterile PBS, and mandibular tissues with tooth germs were micro-dissected from oral commissures. Tissues were incubated at 37 °C for 24 h before medium replacement.

### 2.2 Subcutaneous transplantation of tooth germs

Nude mice were anesthetized via ether inhalation, and their dorsums sterilized. A 1–2 cm paramedian incision was made, subcutaneous tissues dissected, and three experimental groups of tooth germs (control, co-cultured, and mandible EV-treated) were implanted and secured. Wounds were closed in layers. Mice (n = 20) were monitored postoperatively. All animal experiments were conducted in accordance with protocols approved by the Animal Ethics Committee of Shanxi Medical University.

### 2.3 Morphological analysis of tooth germs

After 6 weeks, mice were euthanized by cervical dislocation. Tooth germs were explanted via microdissection and examined under a stereomicroscope (Leica M205 FA) for morphological assessment.

### 2.4 Micro-CT (μCT) analysis

The mineralized portion of the tooth germ was reconstructed and quantified via micro-CT (μCT) scanning. (Quantum GX2, PerkinElmer, U.S). Scanning was performed at 80 kV and 100 μA, with a voxel size of 72 μm and a total acquisition time of 1 min 3D reconstructions and mineralization analyses were performed in Mimics Research 21.0 (Materialise), quantifying surface area, volume, and mineralized volume percentage (MV%).

### 2.5 Isolation and characterization of mandibular extracellular vesicles

Following a 24-h *in vitro* adaptive culture of E40 miniature pig mandibles, the medium was replaced. Conditioned medium was harvested after 72 h and clarified by two rounds of centrifugation (2,000 × g, 20 min each). The resulting supernatant was then combined with an equal volume of Exo-spin™ precipitant and incubated overnight at 4 °C under constant rotation. Subsequently, the mixture was centrifuged at 12,000 × g for 70 min. The final pellet was resuspended in PBS for storage at −80 °C. Isolated EVs underwent morphological characterization via transmission electron microscopy, size distribution analysis by nanoparticle tracking analysis (NTA), and Western blotting for exosomal marker proteins.

### 2.6 Primary cell culture

Primary dental mesenchymal cells from E40 minipig tooth germs were cultured using enzymatic tissue explanation and maintained at 37 °C/5% CO_2_, with medium changes every 48–72 h. Cells exhibiting characteristic spindle/oval morphology with orderly arrangements were passaged at 80%–90% confluence using trypsinization, with passages 3-5 utilized for experiments.

### 2.7 Small RNA sequence

Small RNA sequencing was performed on miRNA extracted from: (1) tooth germs cultured alone (control) and (2) tooth germs exposed to mandible-EVs. Sequencing and analysis were performed by Personalgene Company (Nanjing, China).

### 2.8 qRT-PCR

Following isolation of total RNA from cells using TRIzol reagent, cDNA was generated with a commercial reverse transcription kit (Takara, Japan). Subsequent quantitative real-time PCR (qRT-PCR) amplification analysis was performed on a 7,500 Real-Time PCR system, with gene expression normalization to the GAPDH reference gene. All primers, listed in Supplementary Table S1, were designed, synthesized, and validated by Biotech Company (Changzhou, China).

### 2.9 Western blotting

Western blotting was conducted according to a standard protocol. The total protein was determined by a Pierce BCA Protein Assay Kit (Thermo Fisher Scientific, U.S) using the manufacturer’s instructions. Following quantification, protein separation was achieved using SDS-PAGE, followed by transfer to a membrane, and probed with the following primary antibodies: Rabbit Anti-HDAC4 antibody (1:500, Invitrogen™ 711390, U.S), Rabbit Anti-CD9 antibody (1:1000, Servicebio, GB115697, China), CD63 Rabbit mAb (1: 1000, Servicebio, GB115712, China), ACTIN Rabbit mAb (1:10000 ABclonal, AC038, China).

### 2.10 Luciferase reporter assay

Luciferase reporter assay was performed to validate the direct interaction between miR-206 and HDAC4. Fragments of the HDAC4 3′-UTR, containing either the wild-type (WT) or mutant (MUT) binding sequences, were cloned into pmirGLO vectors (Genema Biotech, China). 293T cells were co-transfected with miR-206 mimics and either the HDAC4-WT or HDAC4-MUT reporter construct. After 48 h, luciferase activities were measured using a dual-luciferase assay system according to the manufacturer’s instructions.

### 2.11 Statistical analysis

Statistical analysis was performed using GraphPad Prism software (version 9.0). Data are expressed as mean ± SD, with inter-group differences determined by one-way ANOVA (normally distributed data with homogeneity of variance) or nonparametric tests (non-normal/variance-heterogeneous data), with statistical significance set at α = 0.05 (*p* < 0.05).

## 3 Results

### 3.1 Mandible-derived extracellular vesicles from miniature pigs regulated early morphogenesis and mineralization of tooth germs

**FIGURE 1:**
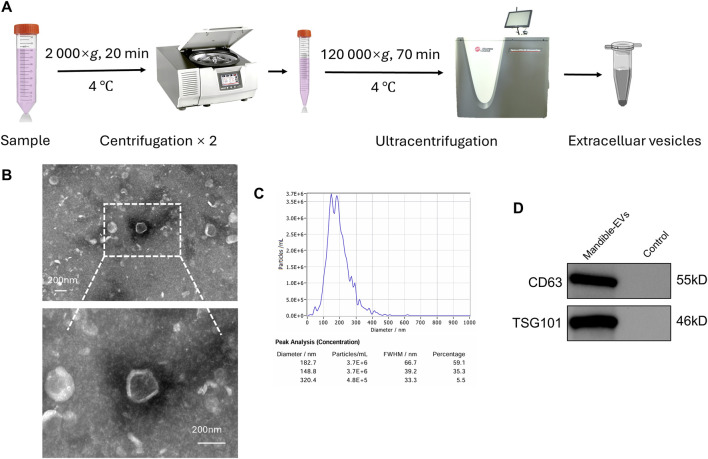
Isolation and biophysical characterization of mandible-derived EVs. **(A)** Workflow for EVs isolation. **(B)** Representative TEM image of negative-staining mandibular EVs. Scale bar: 200 nm. **(C)** Nanoparticle concentration and size profile (NTA). **(D)** Immunoblotting analysis of EVs characteristic markers.

**FIGURE 2:**
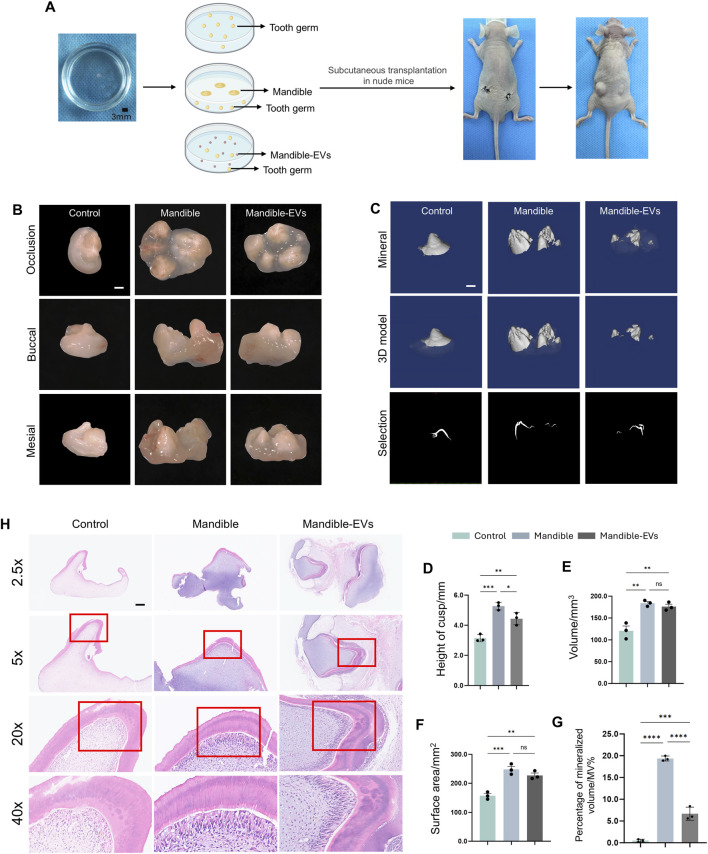
Key role of mandible-derived EVs in odontogenesis and mineralization of tooth germs. **(A)** Schematic of experimental workflow: Tooth germs (exposed to mandible or mandible-EVs) were subjected to subcutaneous transplantation in nude mice. **(B)** Morphology of tooth germs observed under stereomicroscope. Scale bar: 1 mm. **(C)** 3D reconstruction and mineralization analysis of tooth germs by micro-CT. Scale bar: 1 mm. **(D–G)** Quantitative analyses of **(D)** cusp height, **(E)** volume, **(F)** surface area, and **(G)** Percentage of mineralized volume/MV%. **(H)** Histological examination of tooth germ histogenesis by H&E staining. Scale bar: 100 μm *P < 0.05, **P < 0.01. ***P < 0.001. ****P < 0.0001.

We isolated mandibles from E40 miniature pigs at the early bud stage of tooth germs and extracted (EVs) via differential ultracentrifugation (Figure 1A). Physical characterization confirmed typical cup-shaped morphology by transmission electron microscopy (Figure 1B; Supplementary Figures S1A, 2A), with a mean diameter of 179.5 nm measured via nanoparticle tracking analysis (NTA, Figure 1C). Western blotting showed high expression of extracellular vesicles markers CD63 and TSG101 (Figure 1D), confirming EVs isolation. Tooth germs were co-cultured with either mandibular tissue or mandible-derived EVs, using Transwell systems for 7 days, followed by subcutaneous implantation into nude mouse dorsum. Germs were harvested after 6 weeks (Figure 2A). Following harvest, tooth germs underwent three-dimensional morphological assessment via stereomicroscopy and mineralization analysis through micro-CT scanning, with concurrent quantification of surface area, volume and percentage of mineralized volume (Figures 2B–G). Detailed comparative analysis revealed that the control group displayed markedly underdeveloped tooth cusps and a smaller overall surface area compared to both the Mandible and Mandible-EVs groups. Moreover, mineralization was substantially impaired in controls, as evidenced by reduced mineral density and volume fraction in micro-CT reconstructions. We found that the control group exhibited significantly impaired morphogenesis alongside substantially reduced mineralization compared to the other groups. Histological examination by H&E staining further revealed that control specimens exhibited disorganized tissue architecture, with a poorly defined dentin-pulp complex and a complete absence of the distinct, polarized odontoblast layer that was clearly visible lining the predentin in both the Mandible and Mandible-EVs groups. This lack of a structurally integral odontoblast layer in the control group correlates with its observed mineralization defects (Figure 2H). Taken together, these data indicated that extracellular vesicles originating from the mandible are indispensable for tooth germ patterning and biomineralization competence.

### 3.2 Mandible-derived extracellular vesicles delivered miR-206 targeting HDAC4

**FIGURE 3:**
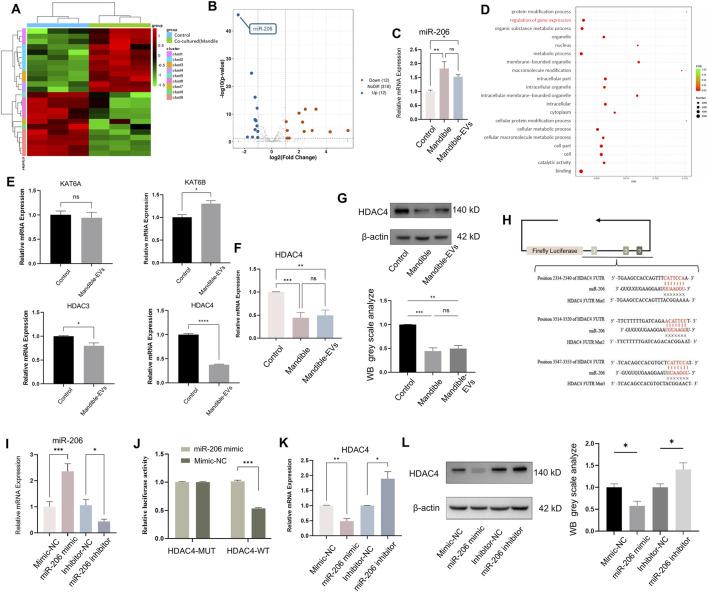
Mandibular EVs transferred miR-206 targeting HDAC4. **(A)** Hierarchical clustering of miRNA expression profiles from tooth germs exposed to mandible-EVs (RNA-seq). **(B)** Volcano analysis highlighted the top 24 candidate miRNAs. **(C)** Quantification of miR-206 in tooth germs. **(D)** GO term enrichment analysis of the tooth germ exposed to mandible-EVs. **(E)** Expression levels of key epigenetic regulators in tooth germs exposed to mandible-EVs. **(F)** Transcriptional expression of HDAC4 (qTR-PCR). **(G)** Translational expression of HDAC4 (Western blotting). **(H)** Design of luciferase reporter construct with HDAC4 3′UTR binding site. **(I)** Quantification of miR-206 upon miR-206 mimic/inhibitor transfection. **(J)** Effect of miR-206 overexpression or inhibition on HDAC4 3′-UTR reporter activity. **(K)** Transcriptional expression of HDAC4 upon miR-206 mimic/inhibitor transfection. **(L)** Translational expression of HDAC4 upon miR-206 mimic/inhibitor transfection. *P < 0.05, **P < 0.01. ***P < 0.001.

To identify critical regulators within mandibular extracellular vesicles, we performed small RNA sequencing (Figure 3A), revealing top 24 differentially expressed miRNAs, with miR-206 showing the most significant alteration (Figure 3B). Subsequent qRT-PCR validation confirmed substantial upregulation of miR-206 in tooth germs (exposed to mandibular tissue and mandible-EVs). (Figure 3C; Supplementary Figures S2, S3). Gene Ontology (GO) analysis of RNA-seq data implicated epigenetic regulators as downstream targets (Figure 3D). We therefore examined four key epigenetic modifiers: histone acetyltransferases (KAT6A, KAT6B) and deacetylases (HDAC3, HDAC4). HDAC4 exhibited the most pronounced differential expression (Figure 3E). Both qRT-PCR and Western blotting confirmed significant downregulation of HDAC4 at RNA and protein levels (Figures 3F,G), establishing HDAC4 as a pivotal epigenetic regulator for subsequent studies. In silico prediction identified three putative miR-206 binding sites located at nucleotides 2334–2340, 3514–3520, and 3547–3553 within the HDAC4 3′UTR (Figure 3H). To validate this regulatory relationship, we co-transfected dental mesenchymal cells with constructed recombinant plasmids alongside miR-206 mimics or negative control mimics (mimics-NC) (Figure 3I). Dual-luciferase assays demonstrated significantly suppressed relative enzymatic activity (*p* < 0.001) in the HDAC4-WT + miR-206 mimics co-transfection group compared to HDAC4-WT + mimics-NC controls, confirming direct targeting of miR-206 to the HDAC4 3′-UTR (Figure 3J). Subsequent validation at transcriptional and translational levels in dental mesenchymal cells revealed that the level of HDAC4 was significantly upregulated following miR-206 inhibition (Figures 3K,L)—mechanistically substantiating that miR-206 negatively regulates HDAC4 through specific 3′-UTR binding.

### 3.3 miR-206/HDAC4 axis regulated early tooth germ morphogenesis and mineralization *in vivo*

**FIGURE 4:**
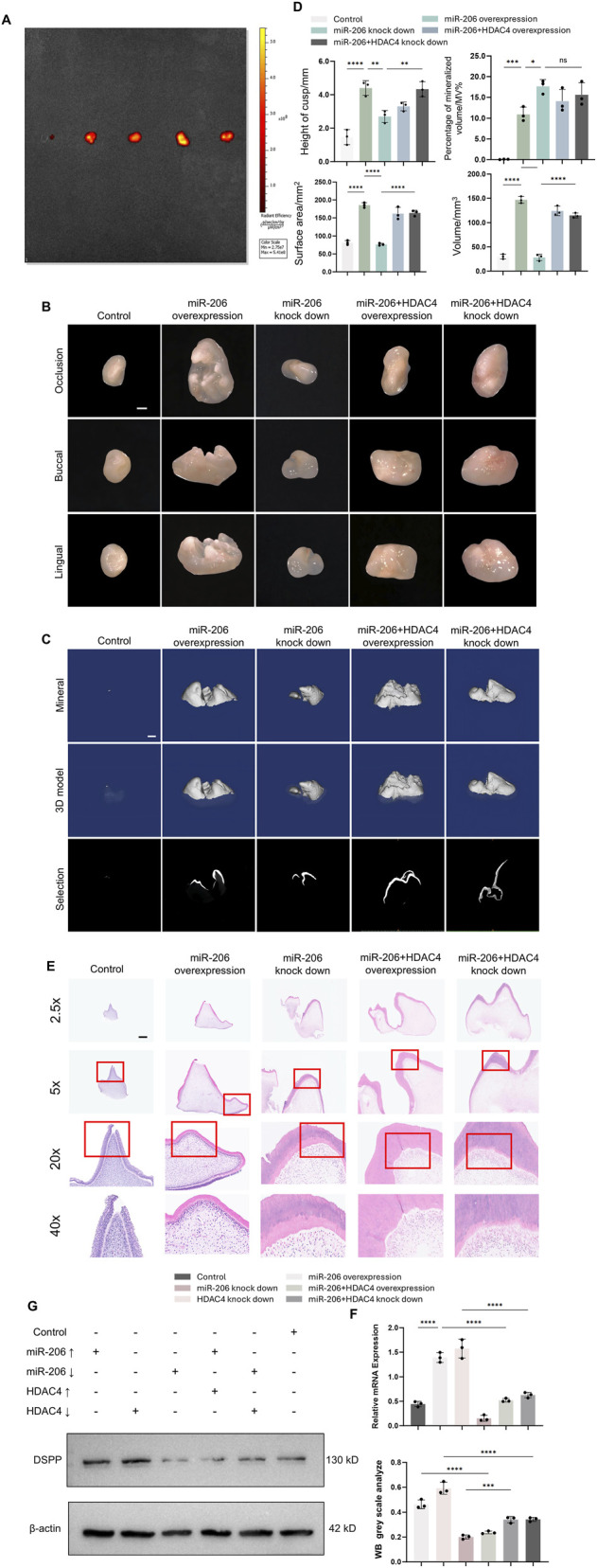
The miR-206/HDAC4 signaling axis regulated early morphogenesis and mineralization of tooth germs. Figure 4. The miR-206/HDAC4 signaling axis governed tooth germ morphogenesis and odontogenic differentiation. **(A)** *In vivo* monitoring of lentiviral transduction in tooth germs via small animal live imaging. **(B)** Morphology of tooth germs observed under stereomicroscope. Scale bar: 1 mm. **(C)** 3D reconstruction and mineralization analysis of tooth germs by micro-CT. Scale bar: 1 mm.**(D)** Quantitative analyses of cusp height, volume, surface area, and Percentage of mineralized volume/MV%. **(E)** Histological examination of tooth germ histogenesis by H&E staining. Scale bar: 100 μm. **(F)** Transcriptional expression of DSPP under miR-206 and HDAC4 regulation. **(G)** Translational expression of DSPP under miR-206 and HDAC4 regulation. *P < 0.05, **P < 0.01. ***P < 0.001. ****P < 0.0001.

**FIGURE 5:**
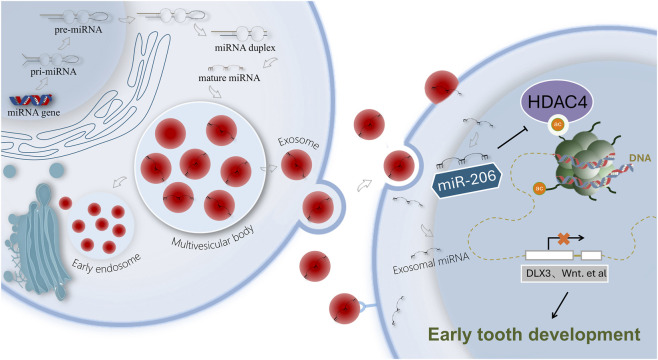
Schematic illustration of the proposed mechanism in this study.

To further assess the functional contribution of the miR-206/HDAC4 axis mechanistically during tooth germ patterning and biomineralization, we transduced tooth germs with lentiviral vectors to overexpress or knockdown miR-206 and HDAC4, followed by subcutaneous implantation into nude mouse dorsum. Viral transduction efficiency was confirmed using small animal *in vivo* imaging, fluorescence microscopy, and qPCR analysis at 6 weeks post-implantation (Figure 4A; Supplementary Figures S3A, 4A–C). Harvested germs underwent stereomicroscopic 3D morphological analysis and micro-CT scanning for mineralization assessment with quantitative measurements of surface area, volume and percentage of mineralized volume. Results demonstrated that miR-206 overexpression yielded morphometric parameters (volume, morphology, mineralization) Quantitative morphometric analyses (Figures 4B–D) further confirmed that tooth germs overexpressing miR-206 exhibited a significant increase in cusp height, surface area, and tissue volume compared to the control group. In contrast, knockdown of miR-206 led to marked reductions in all three parameters, manifesting as flattened cusps, diminished surface area, and reduced volume. Notably, these structural impairments were effectively rescued by concurrent knockdown of HDAC4, which restored cusp height, surface area, and volume to levels comparable to those of the control group (Figures 4B–D). Histological examination further revealed that compared to controls, tooth germs overexpressing miR-206 displayed well-defined dentin matrices, continuous predentin layers, and densely populated odontoblasts exhibiting tightly organized polarized alignment under high magnification (×20, 40x). Conversely, miR-206 knockdown resulted in disoriented odontoblast arrangement with noticeably reduced cell density, aberrant predentin deposition, and poorly mineralized dentin—pathological features observable across all magnifications. Importantly, these defects were substantially reversed in the HDAC4 co-knockdown group, where odontoblast polarity and density were restored, along with the reappearance of a continuous predentin layer and well-mineralized dentin matrix (Figure 4E). To molecularly substantiate the observed morphological improvements in dentin formation, we assessed the expression of dentin sialophosphoprotein (DSPP), a master marker of functional odontoblast differentiation. Consistent with the histological and micro-CT findings, manipulation of the miR-206/HDAC4 axis elicited profound and specific changes in DSPP expression. The pro-differentiation effect of miR-206 overexpression, which mimics mandible EVs, was evidenced by a significant upregulation of DSPP mRNA and protein—a outcome phenocopied by direct HDAC4 knockdown. Critically, rescue experiments solidified the epistatic relationship, demonstrating that whenever DSPP expression was perturbed by miR-206 manipulation (either enhancement by overexpression or impairment by knockdown), it was rescued by a compensatory alteration in HDAC4 levels (Figures 4F,G). Collectively, these genetic interaction data demonstrate that HDAC4 is an essential and primary downstream effector through which miR-206 orchestrates the transcriptional program for odontoblast differentiation and dentin matrix synthesis (Figure 5).

## 4 Discussion

In this study, utilizing an E40 miniature swine model, we isolated mandible-derived extracellular vesicles and demonstrated their regulatory role in early tooth germ development both *in vivo* and *in vitro*. Mechanistically, exosomal miR-206 negatively regulates tooth germ patterning by targeting HDAC4 in tooth germ. This miR-206/HDAC4 signaling axis critically determines early morphogenesis, mineralization extent and odontoblastic differentiation of tooth germs during early tooth development.

Physiologically developed organs arise from spatiotemporally precise gene expression and reciprocal signaling dialogues between donor and recipient tissues (Su et al., 2024; Mattes and Scholpp, 2018). In dentofacial development—a process involving multi-tissue coordination (Luukko and Kettunen, 2014; Thesleff et al., 1995)—cross-talk between tooth and jawbone is indispensable for sustaining the normative morphogenesis of both structures. Alfaqeeh et al. (2013) have found that isolation of the M1 from the surrounding mesenchyme and alveolar bone lead to an expansion of the tooth germ. Li et al. (2018) has suggested that the tooth germ failed to develop properly without essential exosomal cues from the mandible. Takahashi et al. (2019) have shown that the dental follicle (DF), which encases the developing tooth, contains mesenchymal progenitor cells that give rise to the diverse cells of the tooth root-bone interface and orchestrates skeletal tissue formation via parathyroid hormone-related peptide (PTHrP) signaling through its receptor (PPR). Nevertheless, the mechanistic underpinnings of jawbone orchestration of dental development remain incompletely resolved. Our study now identifies the mandible as an active developmental signaling center rather than a passive structural platform. In this study, we proposed a pivotal character of the mandible in early morphogenesis and mineralization extent of tooth germs, establishing its function as a morphogenetic regulatory hub through secretion of extracellular vesicles. Specifically, we demonstrated that mandible spatiotemporally orchestrate tooth development patterning and mineralization via epigenetic cargo delivery.

Owing to their origin from within cells and functional role outside them, extracellular vesicles are often termed “cellular couriers.” This metaphor highlights their capacity to ferry biological cargo across cell boundaries, enabling autocrine or paracrine signaling that modulates physiological processes during tissue development (Pethő et al., 2018). Briefly, Extracellular vesicles (EVs)-mediated intercellular communication enables the transfer of labile molecules—such as miRNAs, proteins, and non-coding RNAs—across tissues while preserving their bioactivity against enzymatic degradation (Dixson et al., 2023). For instance, Jiang et al. (2017) demonstrated that both epithelial and mesenchymal cells generate EVs carrying miR-135a, which epigenetically upregulates dentin matrix protein expression via stimulation of the Wnt/β-catenin signaling cascade. Here, co-culture of tooth germs with mandible-derived EVs resulted in significant downregulation of HDAC4 expression concomitant with morphological alterations in crown patterning—demonstrating that exosomal cargo critically instructs tooth germ development.

The exquisite process of odontogenesis relies on dynamically balanced acetylation levels of key histones—an epigenetic foundation that orchestrates spatiotemporal gene expression during tooth morphogenesis and differentiation (Shvedunova and Akhtar, 2022). In our study, the histone acetylation homeostasis we described constituted a master regulatory switch for odontogenesis—where minor perturbations (HDAC4 dysregulation by mandible-EVs) can cascade into macroscopic morphological alterations. Targeting this equilibrium (via HDAC knocking down) may rescue developmental anomalies rooted in epigenetic imbalance. Intriguingly, our parallel work revealed that co-culture with mandible significantly altered the metabolic profile of tooth germs through untargeted metabolic fingerprinting, with acetyl-CoA emerging as the most differentially abundant metabolite (Supplementary Figure S5, unpublished). As the activated form of the acetyl group, acetyl-CoA engages in various acetylation reactions and is proven to function as a second messenger that modulates histone acetylation. By altering acetylation levels of core histones, it ultimately governs associated gene regulatory cascades (Verdikt and Allard, 2021; Charidemou and Kirmizis, 2024). Critically, we demonstrated that acetyl-CoA flux modulates Dlx-family transcription factors (e.g., Dlx3/Dlx5) through site-specific histone hyperacetylation, thereby directing odontogenic gene expression programs essential for tooth morphogenesis. Collectively, these results underscored histone acetylation homeostasis as a crucial mechanistic determinant in odontogenesis, where precise spatiotemporal control directs tooth germ patterning and mineralization. These discoveries revealed novel mechanisms and candidate therapeutic targets for dental agenesis.

Our *in vivo* and *in vitro* experiments demonstrate that activating the miR-206/HDAC4 axis significantly enhances tooth germ morphogenesis, characterized by thickened dentin matrix, improved mineralization, and polarized columnar morphology of orderly arranged odontoblasts. These morphological improvements fundamentally reflect the activation of the odontoblastic differentiation program—a perspective strongly supported by the coordinated upregulation of the dentin-specific marker DSPP. As a key terminal marker during dentin secretion, the elevated expression of DSPP directly bridges our observed macroscopic morphological changes with cell-specific molecular events, thereby deepening the mechanistic understanding from “morphological regulation” to “cell differentiation control.” This finding aligns with established knowledge: HDAC is a documented key inhibitor of osteo/odontogenic differentiation (Yamauchi et al., 2024; Wang Z. et al., 2025), functioning by maintaining key transcription factors like DLX3 and RUNX2 in an epigenetically silenced state (Saito et al., 2013). Consequently, we propose a coherent working model: mandible-derived exosomes deliver miR-206 to dental mesenchymal cells, specifically suppress HDAC4 expression, and trigger histone acetylation changes. This epigenetic reprogramming relieves the repression on transcription factors such as DLX3/RUNX2, ultimately activating the odontogenic differentiation program, including DSPP expression, which drives dentin matrix secretion and crown morphogenesis. Collectively, this model delineates a novel signaling cascade from inter-tissue communication to cell fate determination.

The miR-206/HDAC4 signaling axis elucidated in this study not only provides a significant new addition to the fundamental biology of tooth development but also highlights its potential translational value in regenerative dentistry. This axis presents a concrete target for guiding odontogenic differentiation by modulating the epigenetic landscape. The discovery of this epigenetic regulatory pathway opens several promising avenues for future investigation: at the molecular level, the complete transcriptional network downstream of this axis warrants further elaboration; at the cellular level, mapping its effects at single-cell resolution would provide unprecedented insights; and developing more precise genetic models could unravel its cell-specific functions. Particularly noteworthy is the metabolic reprogramming observed in our preliminary work, where mandible co-culture induced acetyl-CoA accumulation in tooth germs - providing new insights into the role of metabolism-epigenetics crosstalk in odontogenesis. Pursuing these directions, along with exploring its potential in regenerative scenarios like dentin repair, will establish novel theoretical and practical foundations for tooth regeneration strategies.

## 5 Conclusion

In summary, our identification of the exosomal miR-206/HDAC4 pathway redefined the mandible as an active epigenetic modulator of odontogenesis. This vesicle-mediated regulation enabled long-range delivery of epigenetic effectors—revealing a paradigm shift in craniofacial development and a druggable target for tooth regeneration.

## Data Availability

Data supporting the findings of this study are available within the article and its Supplementary Material. Furthermore, original data are available from the corresponding author on reasonable request.
