# Supplementary material for: Mandibular extracellular vesicles mediate morphogenesis and mineralization of tooth germs in miniature swine through the miR-206/HDAC4 signaling axis
Source: Front Cell Dev Biol. 2026 Jan 5;13:1707072. doi: 10.3389/fcell.2025.1707072 (PMC12813078; doi:10.3389/fcell.2025.1707072)
Supplement: Supplementary file 1 [file DataSheet1.docx]

***Supplementary Material***

**Supplementary Figures**

**
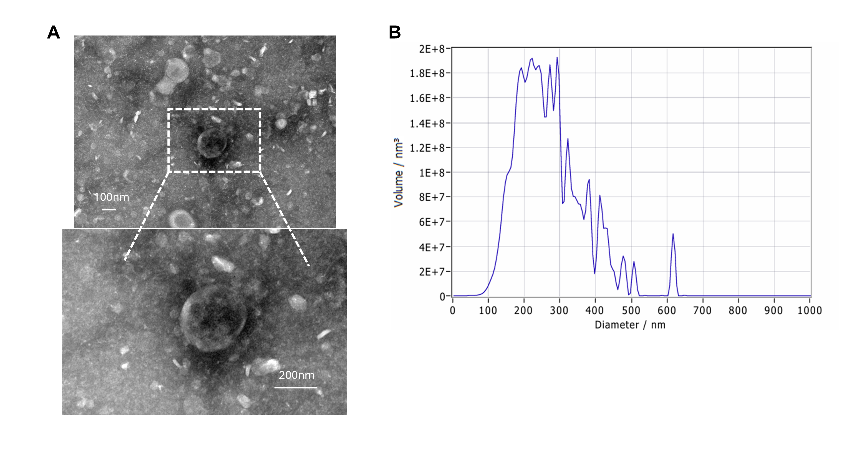
**

**Figure S1: Isolation and characterization of mandible-derived EVs.** (A) Representative transmission electron microscopy (TEM) image of negatively stained mandible-derived EVs. Scale bar: 200 nm. (B) Volume distribution of mandible-derived extracellular vesicles analyzed by nanoparticle tracking analysis (NTA).


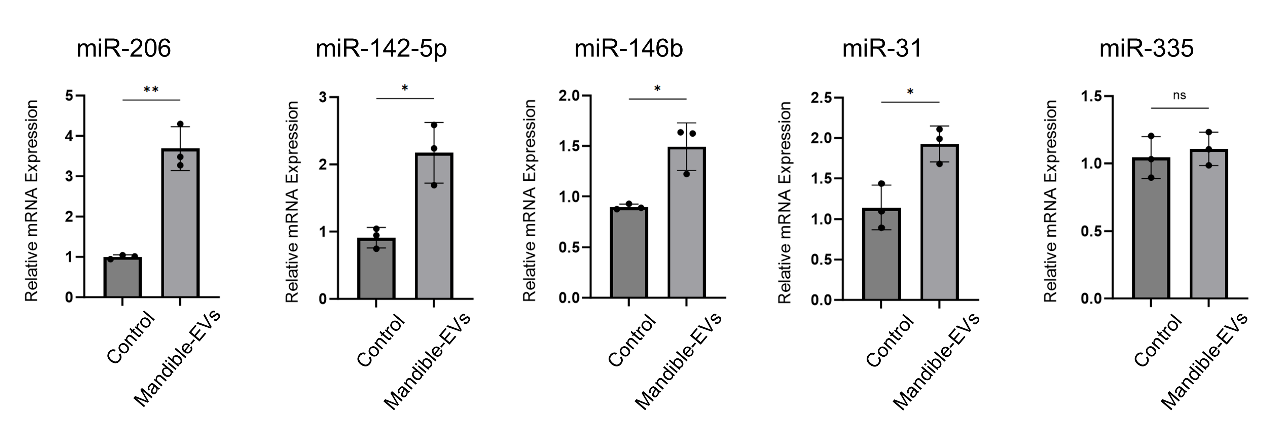


**Figure S2: Mandible-derived EVs delivered key mediator miR-206 to tooth germs.** The level of top 5 differentially upregulated miRNAs identified by RNA-seq. * *P* < 0.05, ** *P* < 0.01.


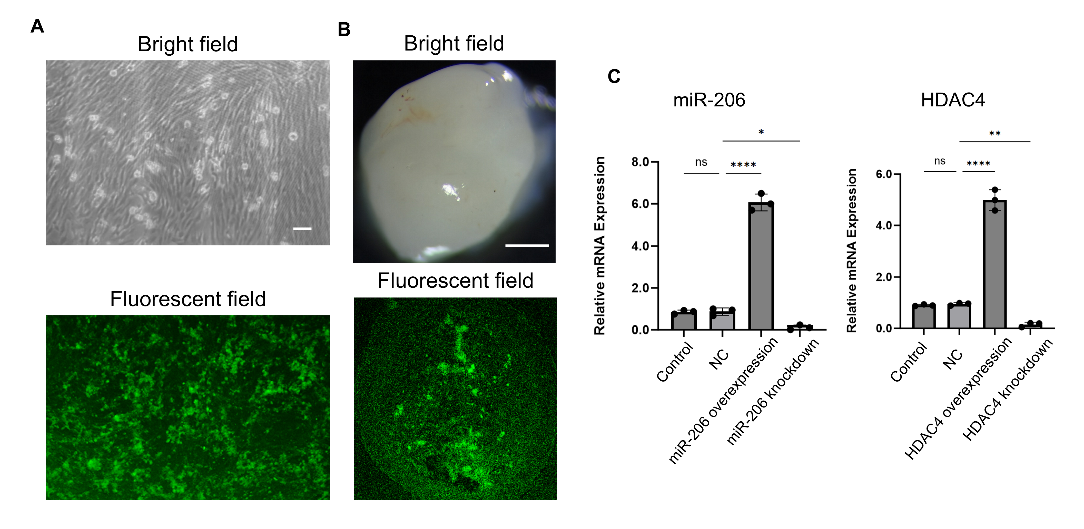


**Figure S3: Lentiviral transduction of tooth germs and dental mesenchymal cells.** (A) miR-206/HDAC4 overexpression/knockdown dental mesenchymal cells were constructed. Scale bar: 100μ m. (B) miR-206/HDAC4 overexpression/knockdown tooth germs were constructed. Scale bar: 1mm. (C) The efficiency of miR-206/HDAC4 overexpression/knockdown. * *P* < 0.05, ** *P* < 0.01, **** *P* < 0.0001.


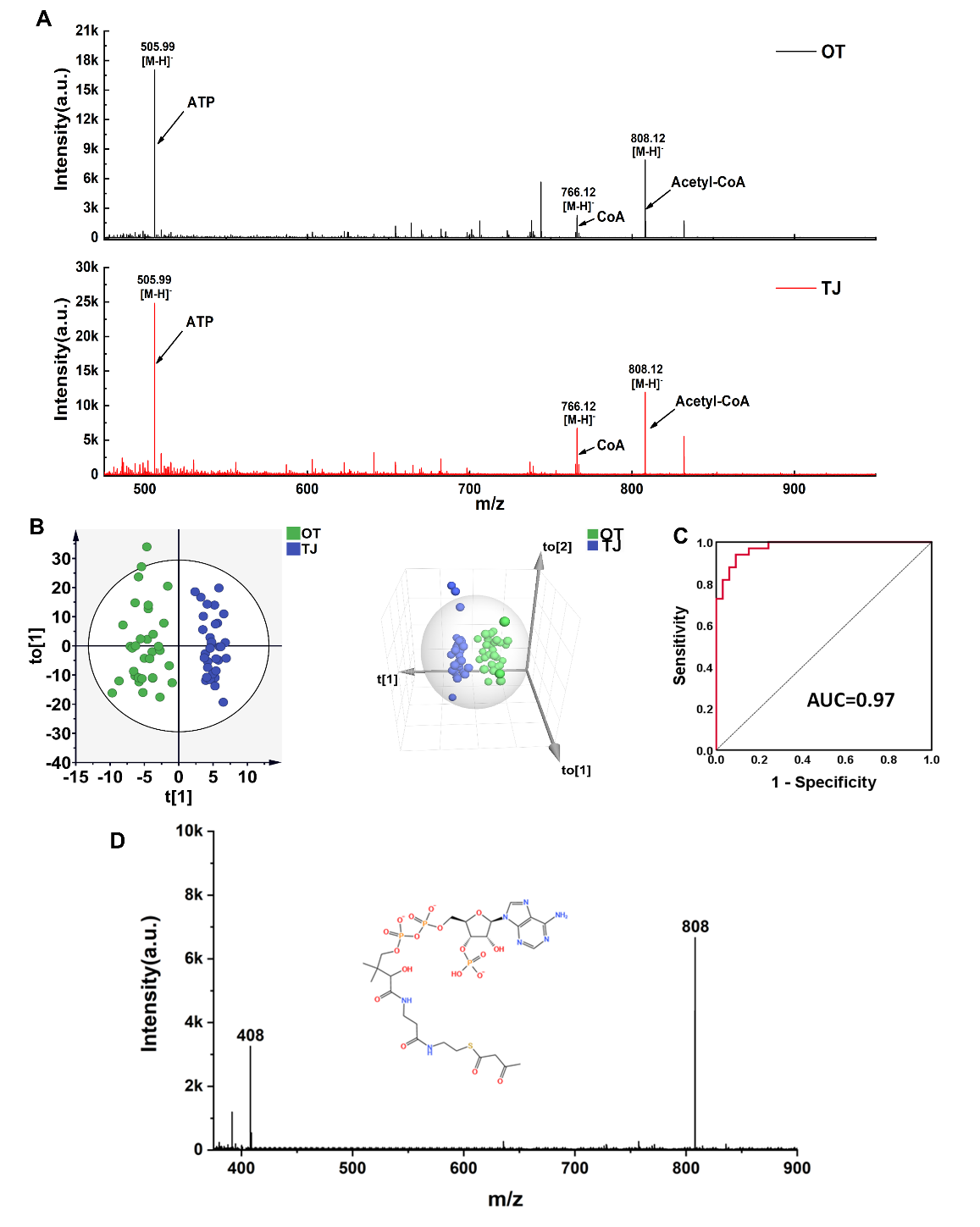


**Figure S4: Mandible-derived signals influenced acetyl-CoA abundance in tooth germs (unpublished).** (A) Untargeted metabolic fingerprinting revealed acetyl-CoA as a prominent differential metabolite in tooth germs exposed to mandible. (B-C). ROC analysis and model evaluation of the untargeted metabolic profiling data. (D). MS/MS spectral confirmation of acetyl-CoA identity.

**Table1** Primer sequences for qRT-PCR.

| **Gene** | **Assay** | | **Forward (5’-3’)** | **Reverse (5’-3’)** |
| --- | --- | --- | --- | --- |
| KAT6B | | RT-qPCR | ACTCAGAGGGGAGCCAGAAT | TGCATTCAGGTGGAGAACATCT |
| KAT6A | | RT-qPCR | TCCTCGCAGCCTCTTGTTTT | GGGACTGCCGAGTTTCATCA |
| HDAC3  HDAC4 | | RT-qPCR  RT-qPCR | TCTTCCAGCCAGTCATCAACC  GAGACCCCTGCCCTCCTC | CGCACTCGCCGTGACCT  AAAGTCCATCTGGGTGGCTC |
| GAPDH | | RT-qPCR | AGGGCTGCTTTTAACTCTGGC | CGTGGGTGGAATCATACTGGA |
| ssc-miR-206 | | RT-qPCR | GCGCGTGGAATGTAAGGAAGT | AGTGCAGGGTCCGAGGTATT |
| U6 | | RT-qPCR | GGAACGATACAGAGAAGATTAGC | TGGAACGCTTCACGAATTTGCG |
